# Supplementary figures and images for: TNF-α exacerbates SARS-CoV-2 infection by stimulating CXCL1 production from macrophages
Source: PLoS Pathog. 2024 Dec 9;20(12):e1012776. doi: 10.1371/journal.ppat.1012776 (PMC11658697; doi:10.1371/journal.ppat.1012776)

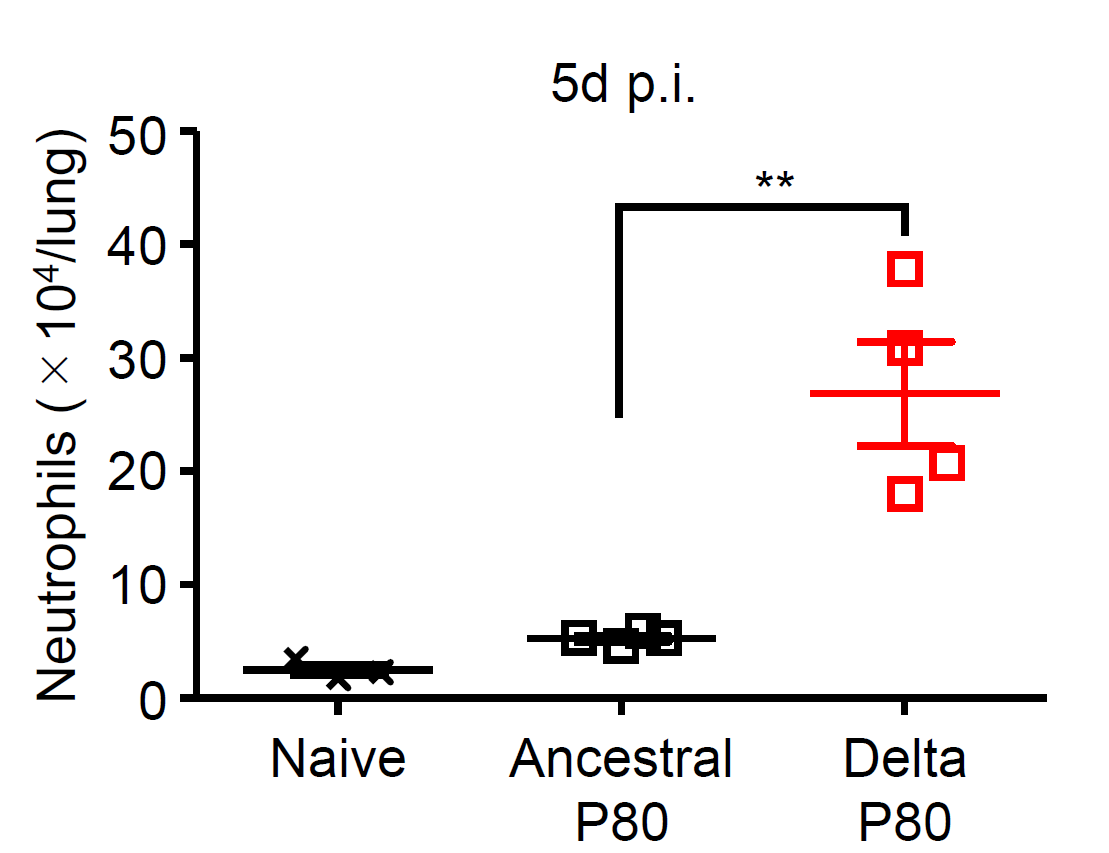

Supplement: S1 Fig — Six-week-old C57BL/6 mice were infected intranasally with 1×10^5^ pfu of the ancestral or Delta P80 virus. Five days later, leukocytes were isolated from the lung. The number of Ly6C+ Ly6G+ neutrophils were analyzed by flow cytometry. Each symbol indicates individual values. Statistical significance was analyzed by two-way analysis of variance (ANOVA). **P < 0.01. (TIF) [file ppat.1012776.s001.tif]

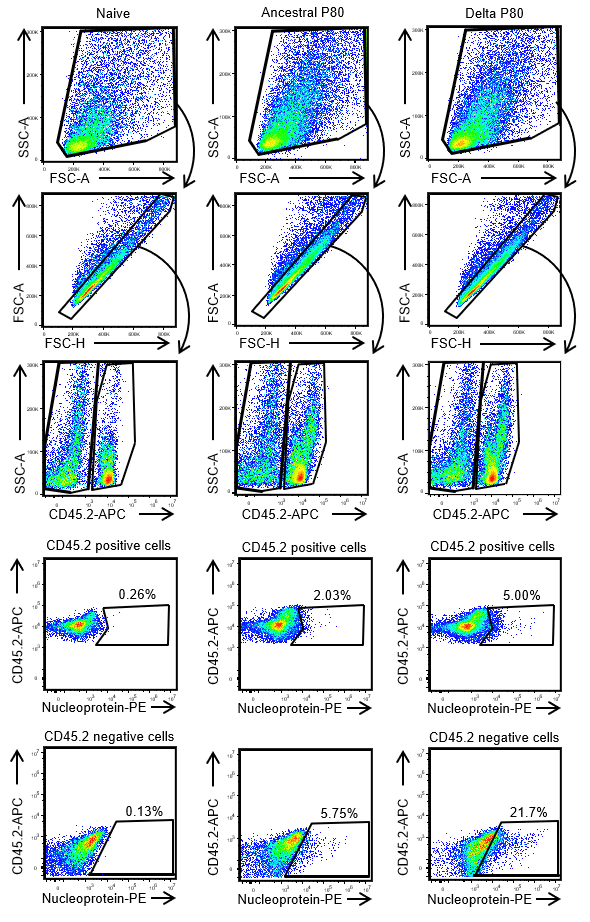

Supplement: S2 Fig — Six-week-old C57BL/6 mice were infected intranasally with 1×10^5^ pfu of the ancestral or Delta P80 virus. Leukocytes were isolated from the lung at 3 days post infection, and intracellularly stained with the nucleoprotein-specific antibody. The frequency of the SARS-CoV-2-infected CD45.2+ or CD45.2– cells were analyzed by flow cytometry. (TIF) [file ppat.1012776.s002.tif]

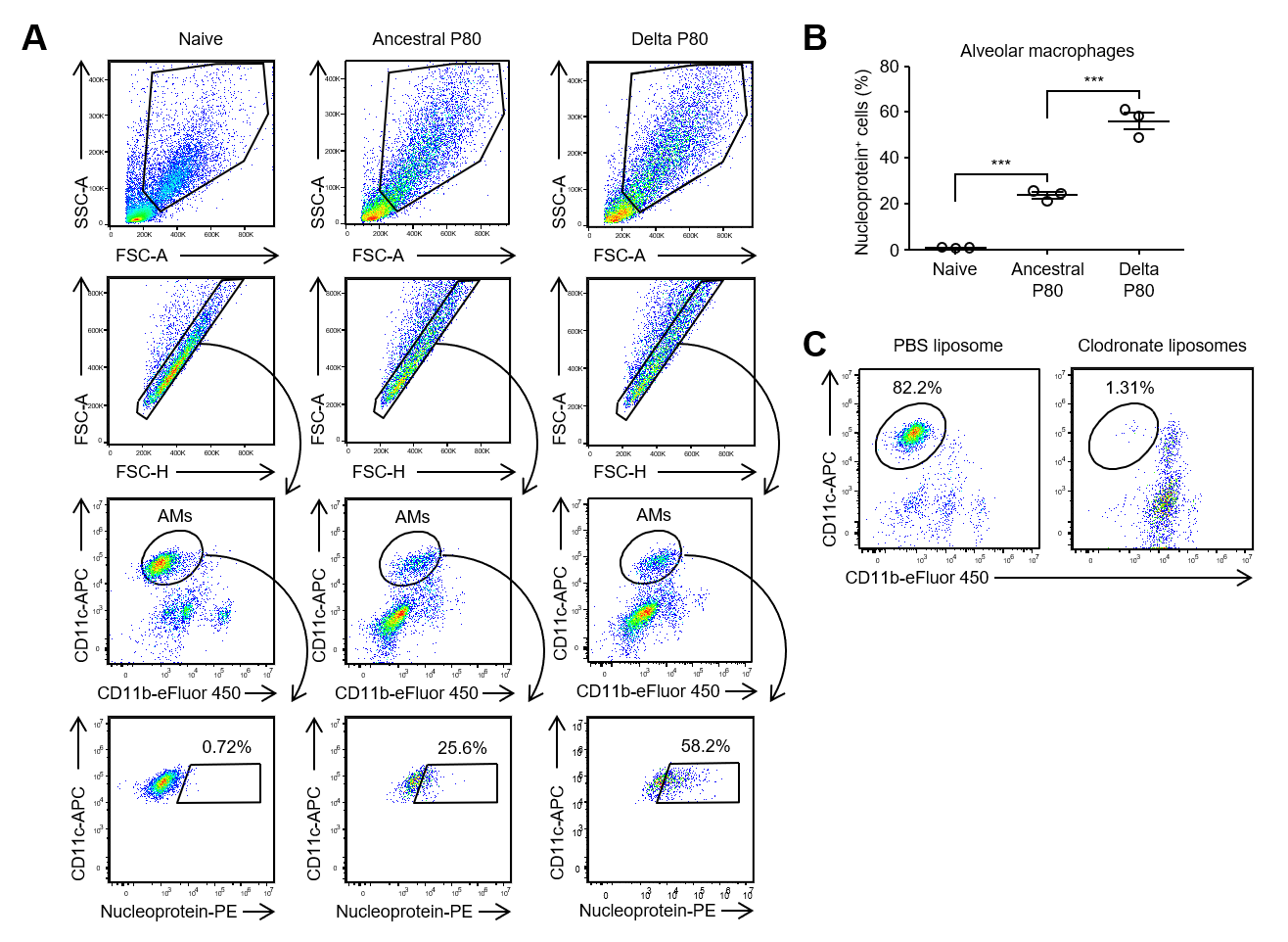

Supplement: S3 Fig — (A and B) Six-week-old C57BL/6 mice were infected intranasally with 1×10^5^ pfu of the ancestral or Delta P80 virus. Leukocytes were isolated from the lung washes at 3 days post infection, and intracellularly stained with the nucleoprotein-specific antibody. To identify alveolar macrophage population, leucocytes were gated by forward and side scatter. Doublet signals are excluded by plotting forward scatter area versus forward scatter height. CD11b and CD11c double-positive cells were identified as alveolar macrophages (A). Frequency of nucleoprotein+ cells in alveolar macrophages are shown (B). (C) The population was confirmed to be macrophages by depletion with clodronate liposomes. Each symbol indicates individual values. Statistical significance was analyzed by two-way analysis of variance (ANOVA) (B). ***P < 0.001. (TIF) [file ppat.1012776.s003.tif]

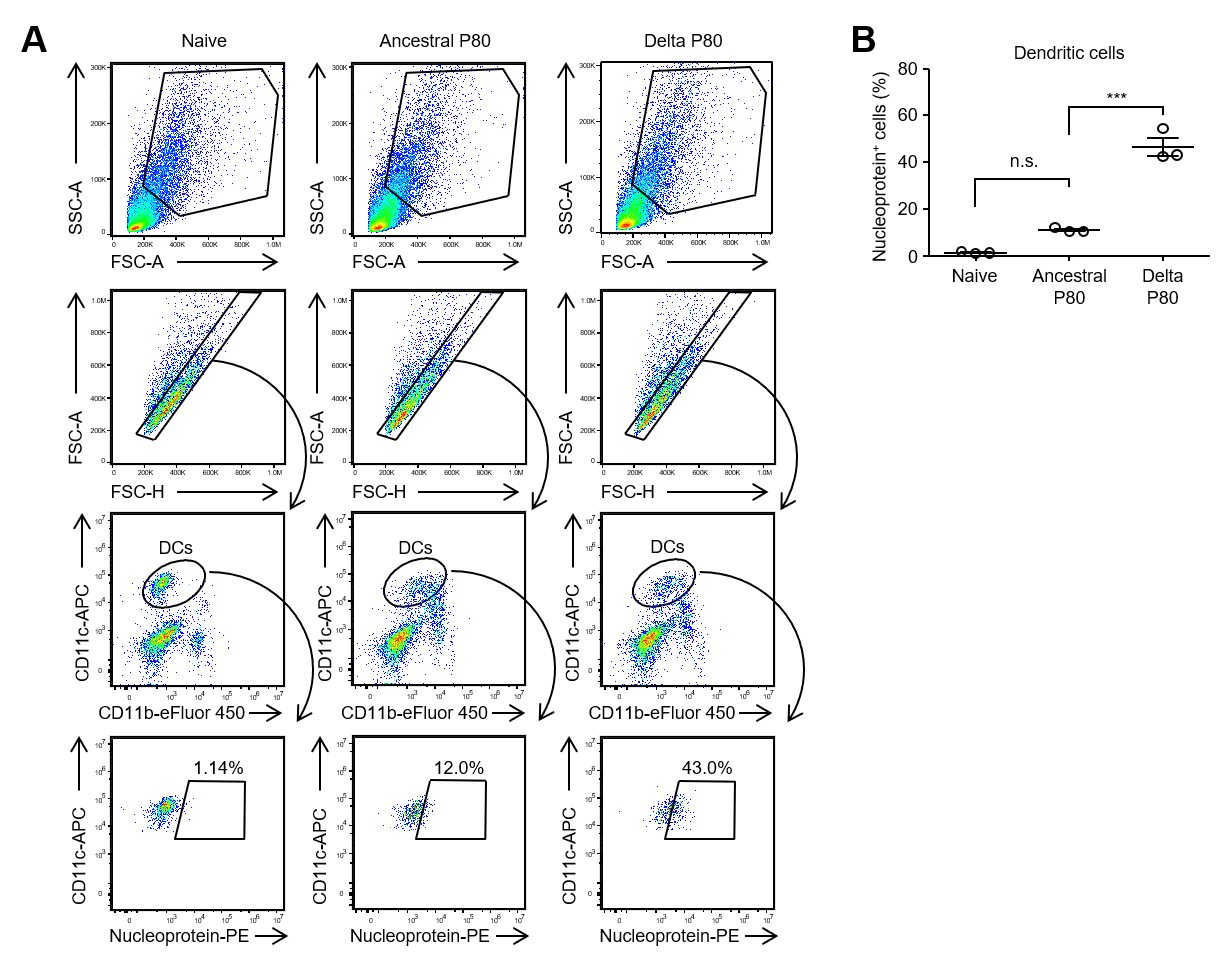

Supplement: S4 Fig — (A and B) Six-week-old C57BL/6 mice were infected intranasally with 1×10^5^ pfu of the ancestral or Delta P80 virus. Leukocytes were isolated from the lung at 3 days post infection, and intracellularly stained with the nucleoprotein-specific antibody. To identify dendritic cell population, leucocytes were gated by forward and side scatter. Doublet signals are excluded by plotting forward scatter area versus forward scatter height. CD11c-positive cells were identified as dendritic cells (A). Frequency of nucleoprotein+ cells in CD11c+ dendritic cells are shown (B). Each symbol indicates individual values. Statistical significance was analyzed by two-way analysis of variance (ANOVA) (B). ***P < 0.001, n.s., not significant. (TIF) [file ppat.1012776.s004.tif]

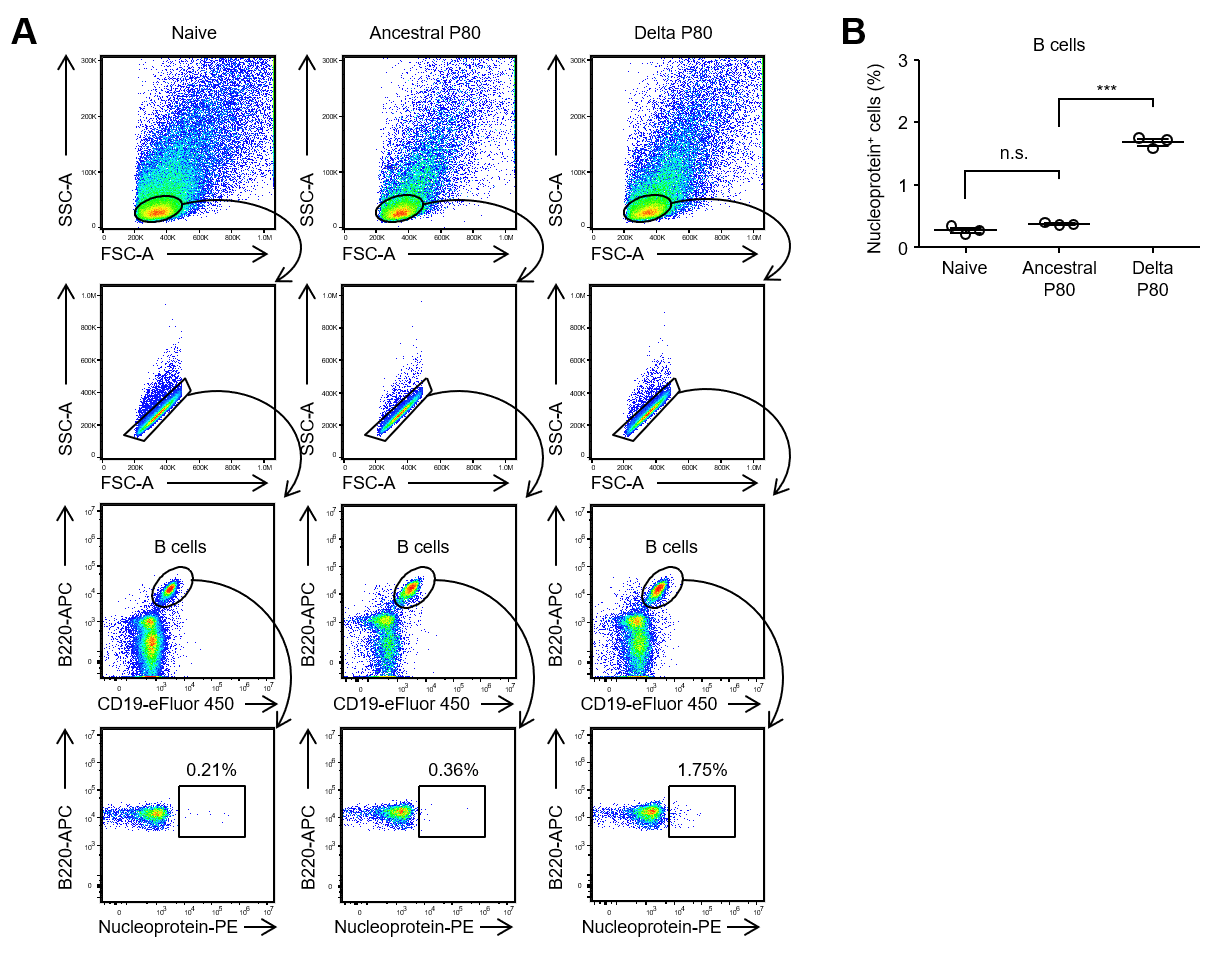

Supplement: S5 Fig — (A and B) Six-week-old C57BL/6 mice were infected intranasally with 1×10^5^ pfu of the ancestral or Delta P80 virus. Leukocytes were isolated from the lung at 3 days post infection, and intracellularly stained with the nucleoprotein-specific antibody. To identify B cell population, leucocytes were gated by forward and side scatter. Doublet signals are excluded by plotting forward scatter area versus forward scatter height. CD19 and B220 double-positive cells were identified as B cells (A). Frequency of nucleoprotein+ cells in B cells are shown (B). Each symbol indicates individual values. Statistical significance was analyzed by two-way analysis of variance (ANOVA) (B). ***P < 0.001, n.s., not significant. (TIF) [file ppat.1012776.s005.tif]

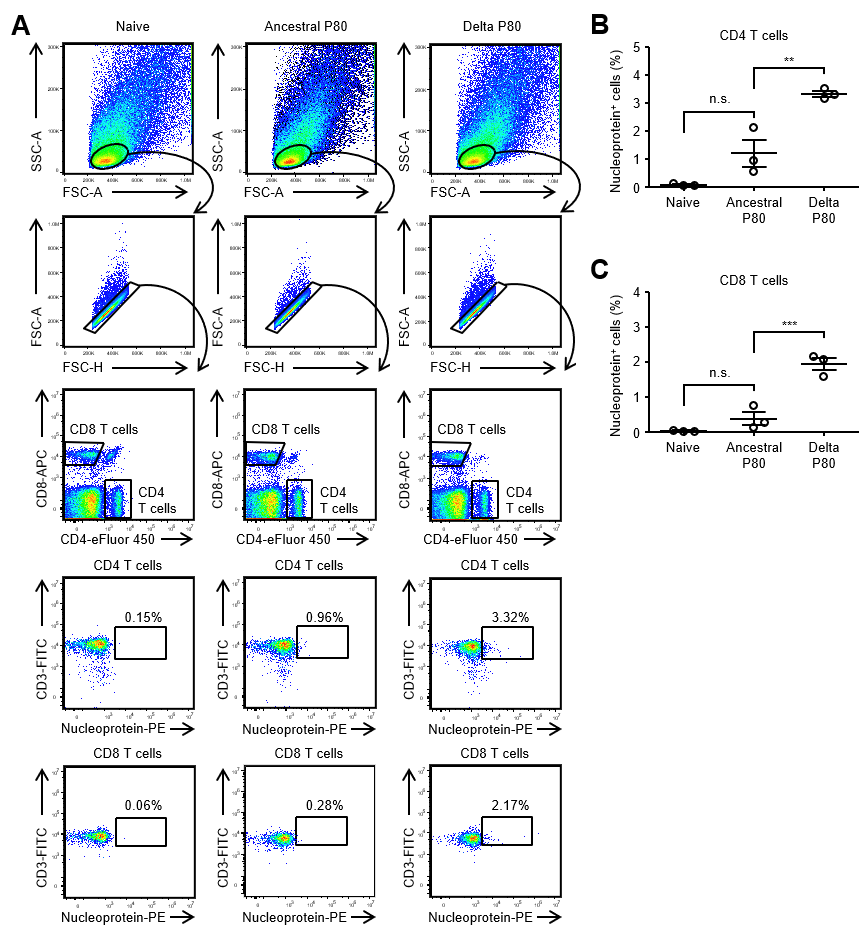

Supplement: S6 Fig — (A-C) Six-week-old C57BL/6 mice were infected intranasally with 1×10^5^ pfu of the ancestral or Delta P80 virus. Leukocytes were isolated from the lung at 3 days post infection, and intracellularly stained with the nucleoprotein-specific antibody. To identify CD4+ and CD8+ T cell population, leucocytes were gated by forward and side scatter. Doublet signals are excluded by plotting forward scatter area versus forward scatter height. CD3 and CD4 or CD3 and CD8 double-positive cells were identified as CD4+ and CD8+ T cells, respectively (A). Frequency of nucleoprotein+ cells in CD4+ (B) and CD8+ T cells (C) are shown. Each symbol indicates individual values. Statistical significance was analyzed by two-way analysis of variance (ANOVA) (B and C). **P < 0.01, ***P < 0.001, n.s., not significant. (TIF) [file ppat.1012776.s006.tif]

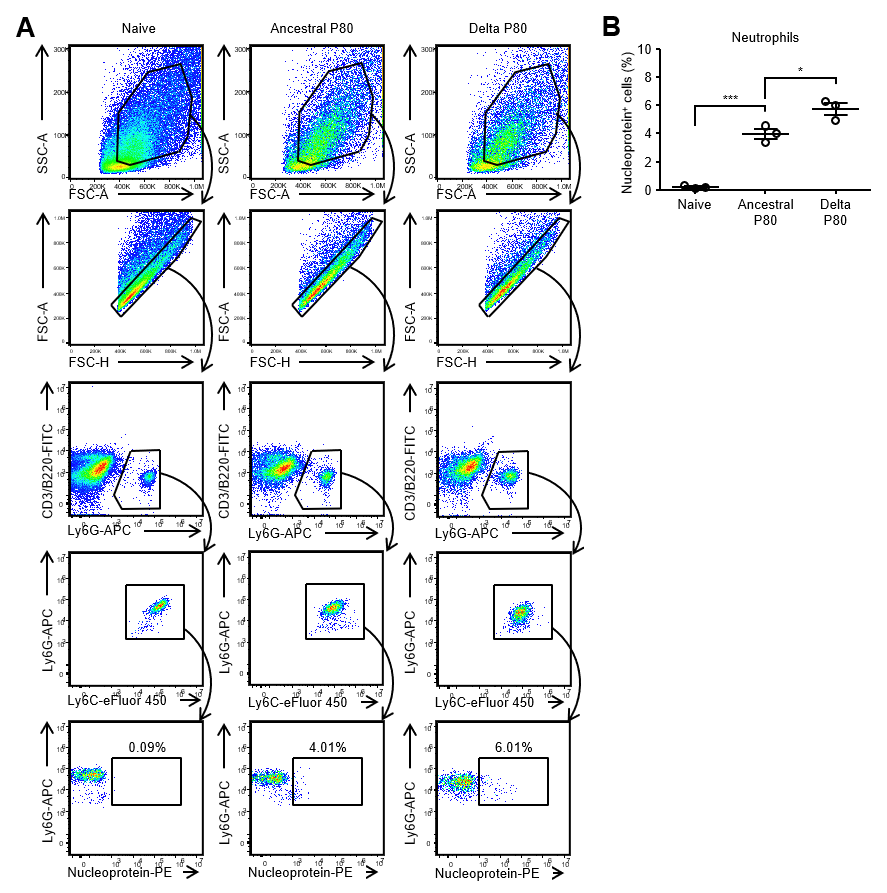

Supplement: S7 Fig — (A and B) Six-week-old C57BL/6 mice were infected intranasally with 1×10^5^ pfu of the ancestral or Delta P80 virus. Leukocytes were isolated from the lung at 3 days post infection, and intracellularly stained with the nucleoprotein-specific antibody. To identify neutrophil population, leucocytes were gated by forward and side scatter. Doublet signals are excluded by plotting forward scatter area versus forward scatter height. Then, B cells and T cells were excluded based on B220 and CD3 expression, respectively. Ly6C and Ly6G double-positive cells were identified as neutrophils (A). Frequency of nucleoprotein+ cells in neutrophils are shown (B). Each symbol indicates individual values. Statistical significance was analyzed by two-way analysis of variance (ANOVA) (B). *P < 0.05, ***P < 0.001. (TIF) [file ppat.1012776.s007.tif]

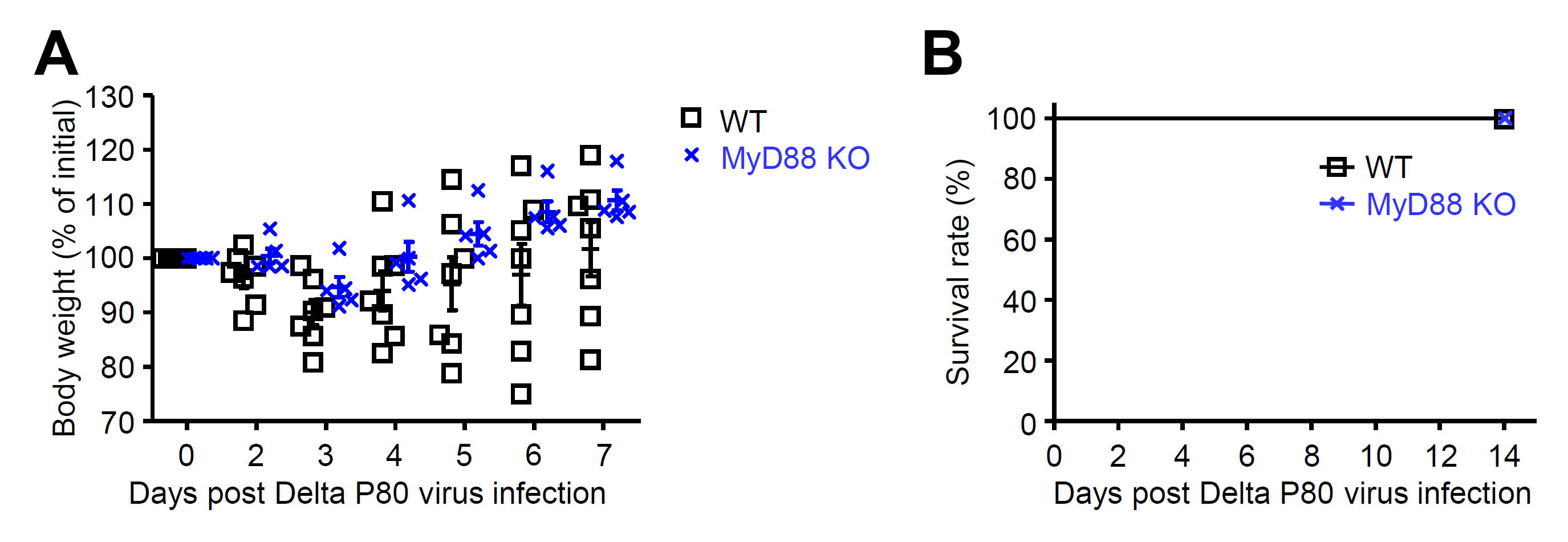

Supplement: S8 Fig — (A and B) Six-week-old C57BL/6 WT or MyD88 mice were infected intranasally with 1×10^4^ pfu of the Delta P80 virus. Weight loss (A) and mortality (B) were monitored for 14 days. Statistical significance was analyzed by two-tailed unpaired Student’s t test (A) or two-sided log-rank (Mantel-Cox) test (B). (TIF) [file ppat.1012776.s008.tif]

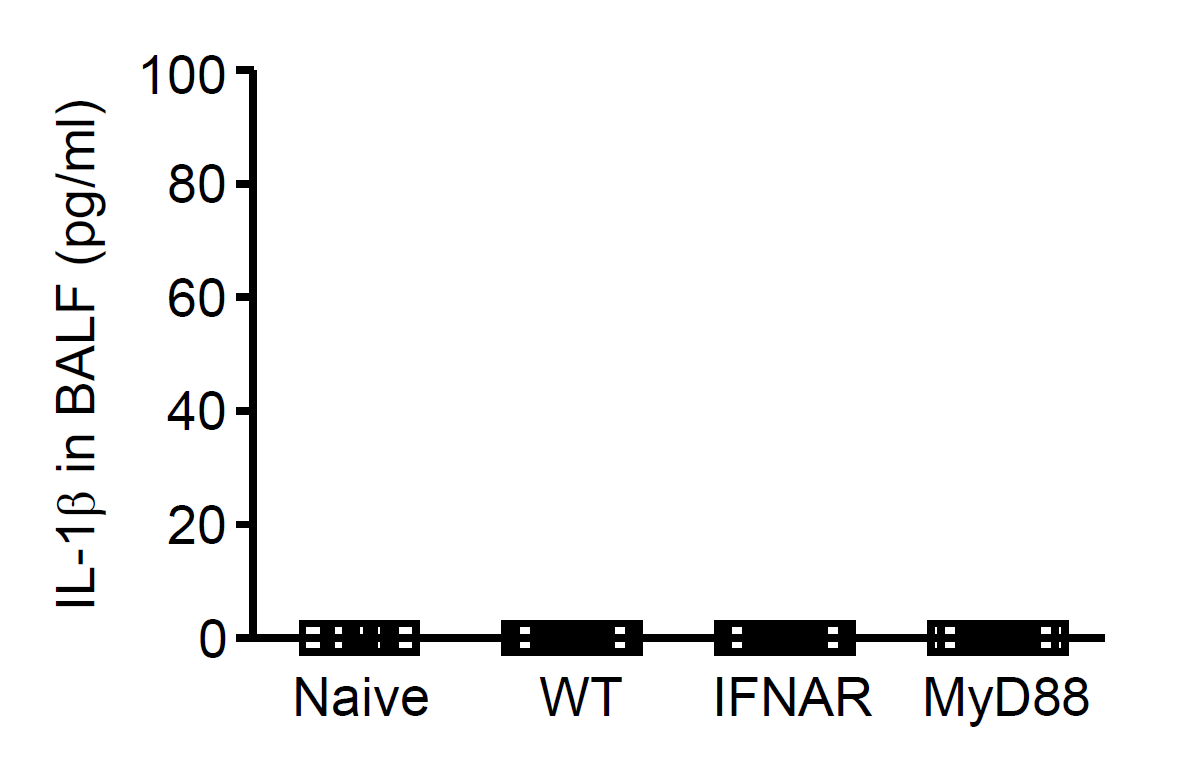

Supplement: S9 Fig — C57BL/6 WT, MyD88, or IFNAR1 KO mice were infected intranasally with 1×10^5^ pfu of the Delta P80 virus. The lung washes were collected at 2 days p.i. and analyzed for IL-1β by ELISA. Each symbol indicates individual values. (TIF) [file ppat.1012776.s009.tif]

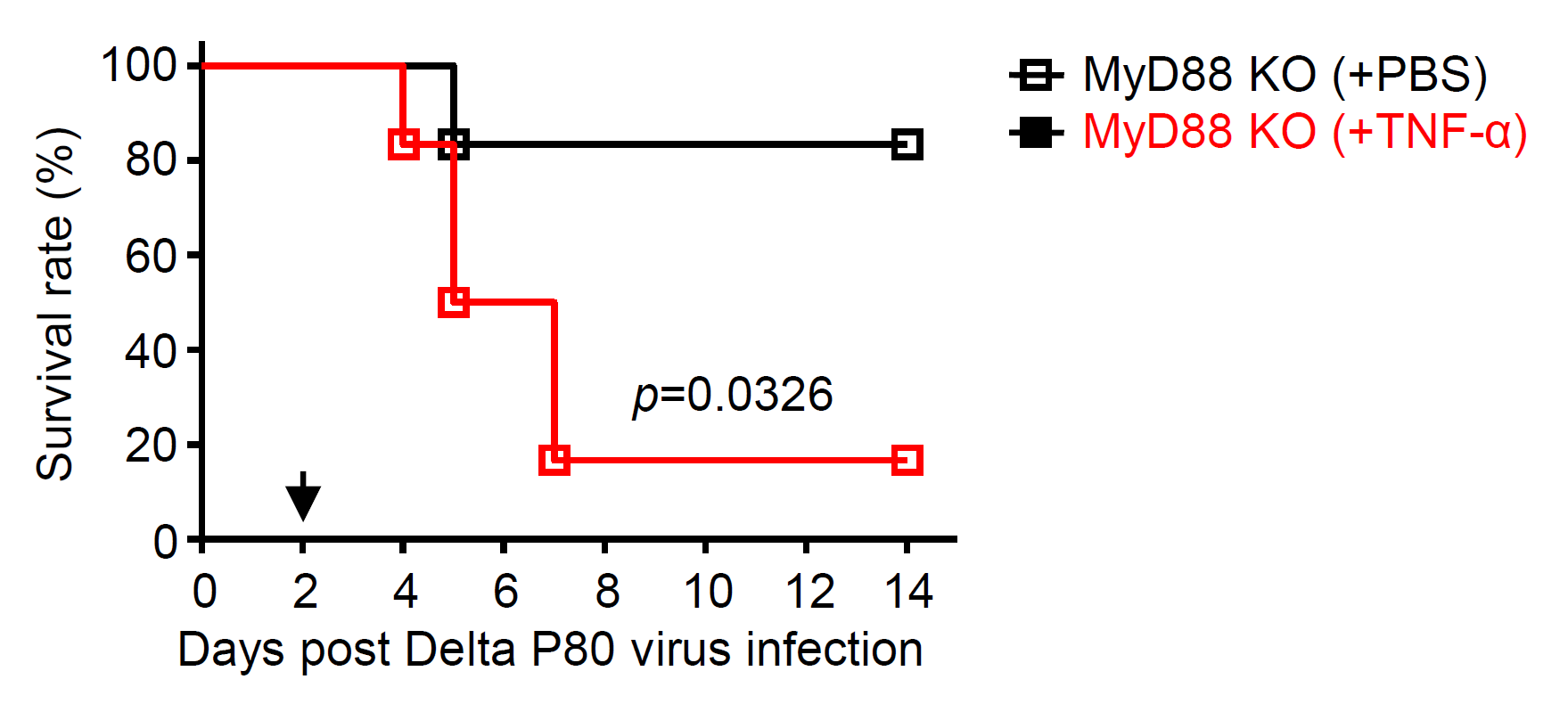

Supplement: S10 Fig — Six-week-old MyD88 KO mice infected with the Delta P80 virus were administered intranasally with PBS or recombinant mouse TNF-α (2.5 μg) at 2 days p.i. (arrow). Mortality was monitored for 14 days. Statistical significance was analyzed by two-sided log-rank (Mantel-Cox) test. (TIF) [file ppat.1012776.s010.tif]

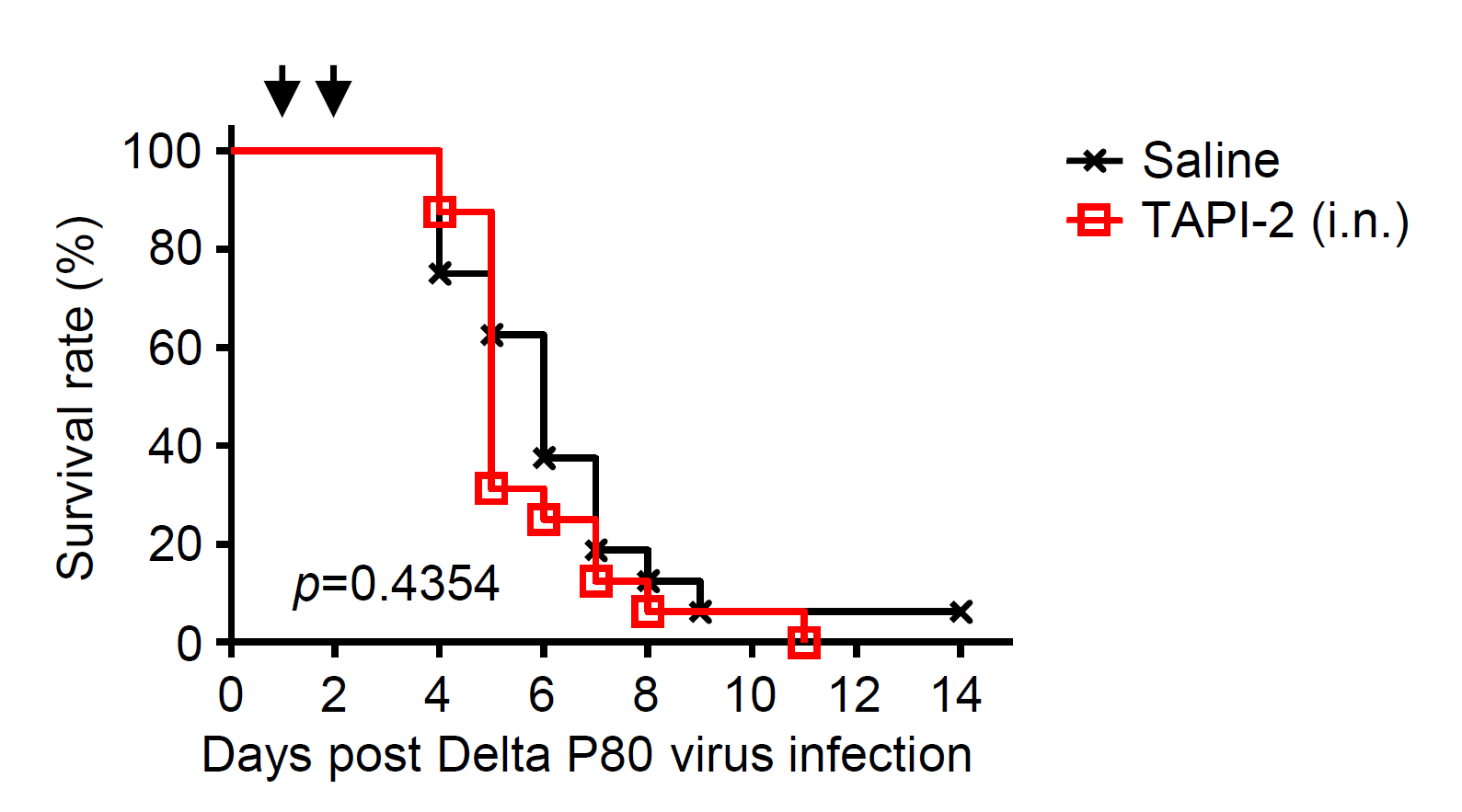

Supplement: S11 Fig — Six-week-old C57BL/6 mice infected with 1×10^5^ pfu of the Delta P80 virus were administered intranasally with saline or TAPI-2 (0.5 μg) at 1 and 2 days p.i. (allow). Mortality was monitored for 14 days. Statistical significance was analyzed by two-sided log-rank (Mantel-Cox) test. (TIF) [file ppat.1012776.s011.tif]

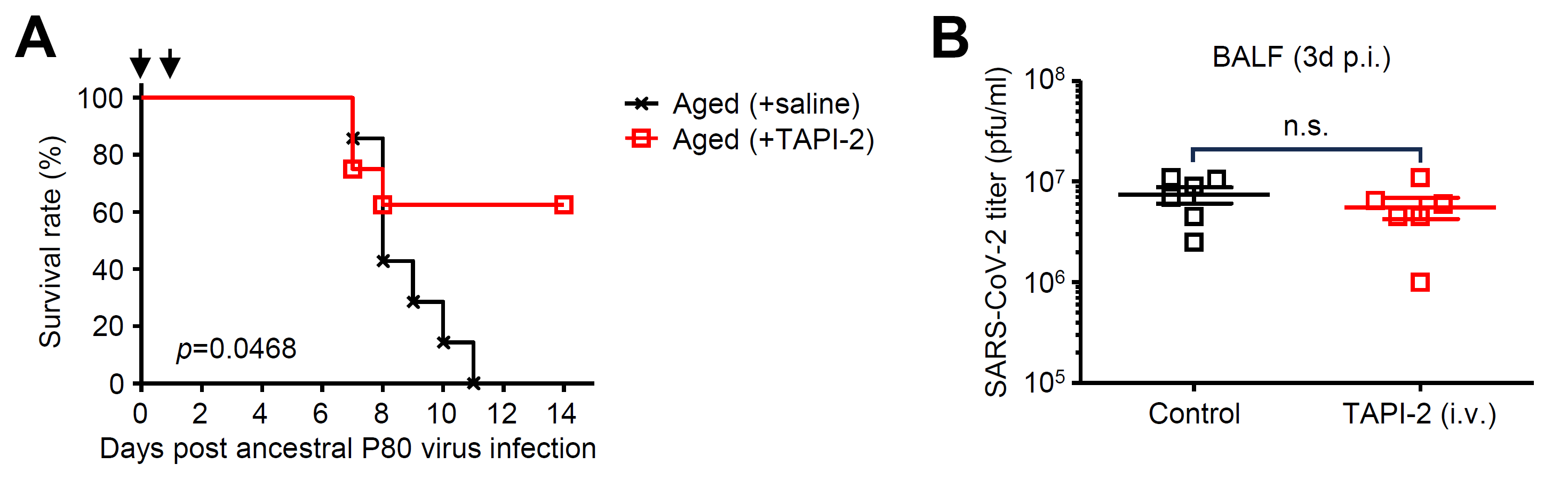

Supplement: S12 Fig — (A and B) Aged (21-week-old) C57BL/6 mice were infected intranasally with 100 pfu of the ancestral P80 virus. Then, infected mice were administered intravenously with saline or TAPI-2 (2.5 μg) at indicated time points (arrows). Mortality was monitored for 14 days (A). The lung washes were collected at 3 days p.i. and viral titers were determined by standard plaque assay (B). Each symbol indicates individual values (B). Statistical significance was analyzed by two-sided log-rank (Mantel-Cox) test (A), or two-tailed unpaired Student’s t test (B). n.s., not significant. (TIF) [file ppat.1012776.s012.tif]

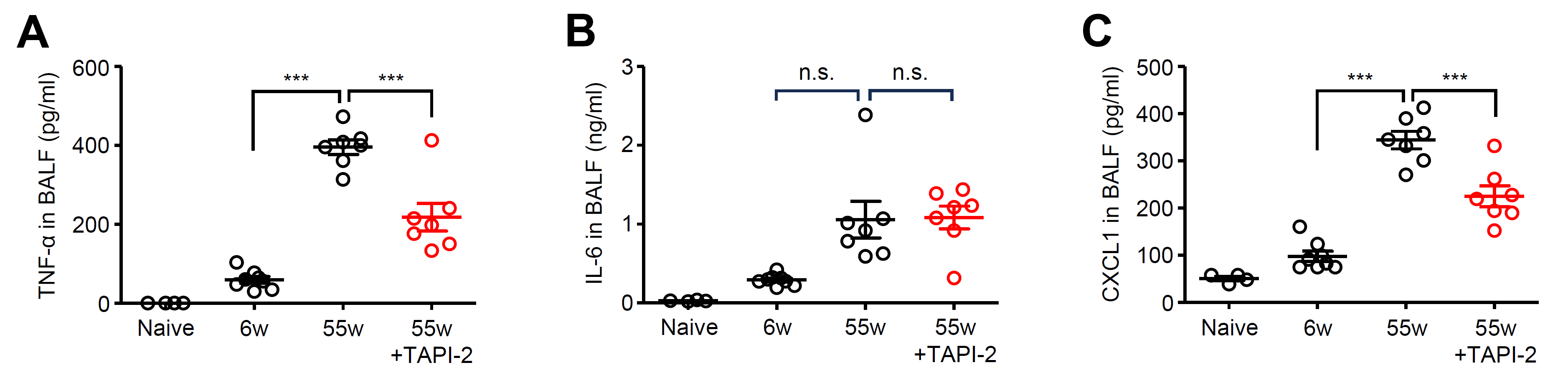

Supplement: S13 Fig — (A-C) Aged (55-week-old) C57BL/6 mice were infected intranasally with 1×10^5^ pfu of the Delta P80 virus. Then, infected mice were administered intravenously with saline or TAPI-2 (2.5 μg) at 0, 1, and 2 days p.i.. The lung washes were collected at 2 days p.i. and analyzed for TNF-α (A), IL-6 (B), or CXCL1 (C) by ELISA. Each symbol indicates individual values. Statistical significance was analyzed by two-way analysis of variance (ANOVA). ***P < 0.001, n.s., not significant. (TIF) [file ppat.1012776.s013.tif]

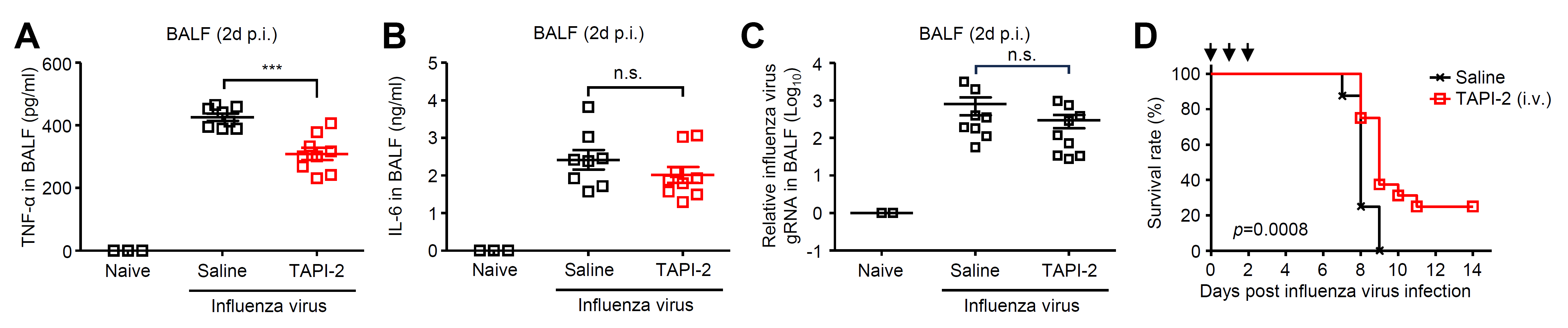

Supplement: S14 Fig — Six-week-old C57BL/6 mice infected with 1×10^3^ pfu of the PR8 virus were administered intravenously with saline or TAPI-2 (2.5 μg) at 0, 1, and 2 days p.i. (allow). (A-C) The lung washes were collected at 2 days p.i. and analyzed for TNF-α (A) or IL-6 (B) by ELISA. Total RNAs were extracted from lung washes and influenza virus NP RNA levels were assessed by quantitative reverse transcription PCR (C). (D) Mortality was monitored for 14 days. Each symbol indicates individual values (A-C). Statistical significance was analyzed by two-tailed unpaired Student’s t test (A-C), or two-sided log-rank (Mantel-Cox) test (D). ***P < 0.001, n.s., not significant. (TIF) [file ppat.1012776.s014.tif]

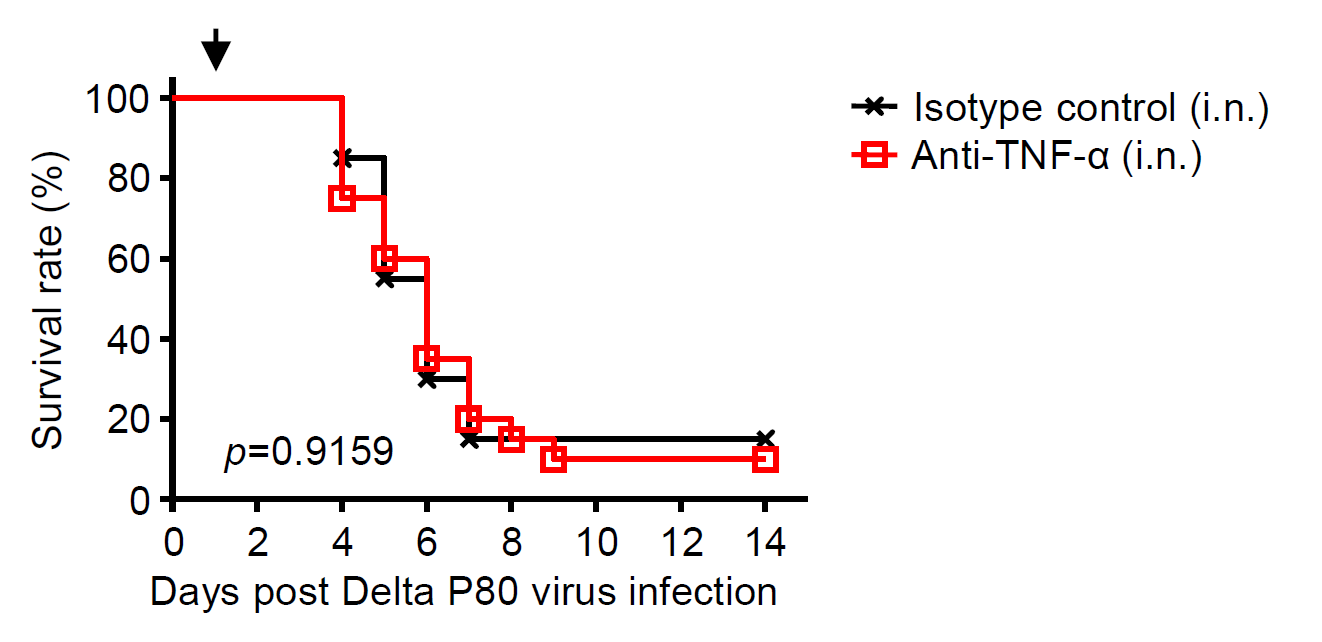

Supplement: S15 Fig — Six-week-old C57BL/6 mice infected with 1×10^5^ pfu of the Delta P80 virus were administered intranasally with isotype rat IgG (2.5 μg) or anti-TNF-α antibodies (2.5 μg) at 1 day p.i. (allow). Mortality was monitored for 14 days. Statistical significance was analyzed by two-sided log-rank (Mantel-Cox) test. (TIF) [file ppat.1012776.s015.tif]

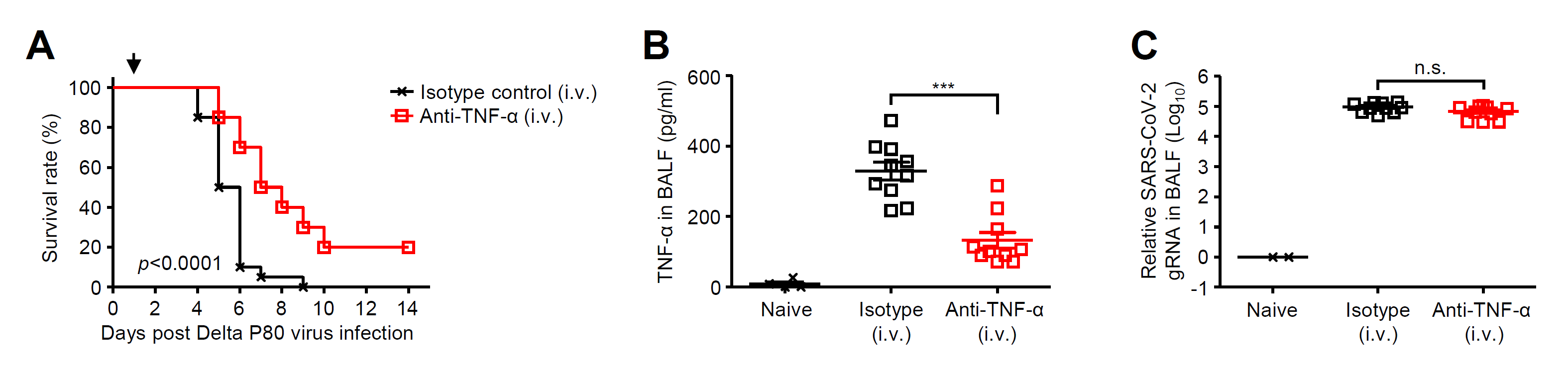

Supplement: S16 Fig — (A-C) Six-week-old C57BL/6 mice infected with 1×10^5^ pfu of the Delta P80 virus were administered intravenously with isotype rat IgG (10 μg) or anti-TNF-α antibodies (10 μg) at 1-day p.i. (allow). Mortality was monitored for 14 days (A). The lung washes were collected at 2 days p.i. and analyzed for TNF-α by ELISA (B). Total RNAs were extracted from lung washes and SARS-CoV-2 N gRNA levels were assessed by quantitative reverse transcription PCR (C). Each symbol indicates individual values (B and C). Statistical significance was analyzed by two-sided log-rank (Mantel-Cox) test (A), two-way analysis of variance (ANOVA) (B), or two-tailed unpaired Student’s t test (C). ***P < 0.001, n.s., not significant. (TIF) [file ppat.1012776.s016.tif]

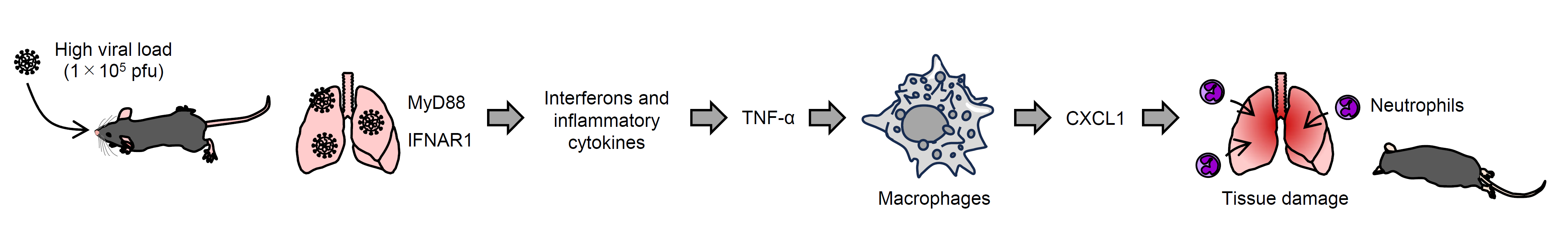

Supplement: S17 Fig — Infection with a lethal dose (1×10^5^ pfu) of the Delta P80 virus enhances type I IFNs and proinflammatory cytokines production in a MyD88- and IFNAR1-dependent manner. TNF-α stimulates CXCL1 production from macrophages, which may enhance lung tissue damage by neutrophils and the disease severity following the Delta P80 virus infection. (TIF) [file ppat.1012776.s017.tif]

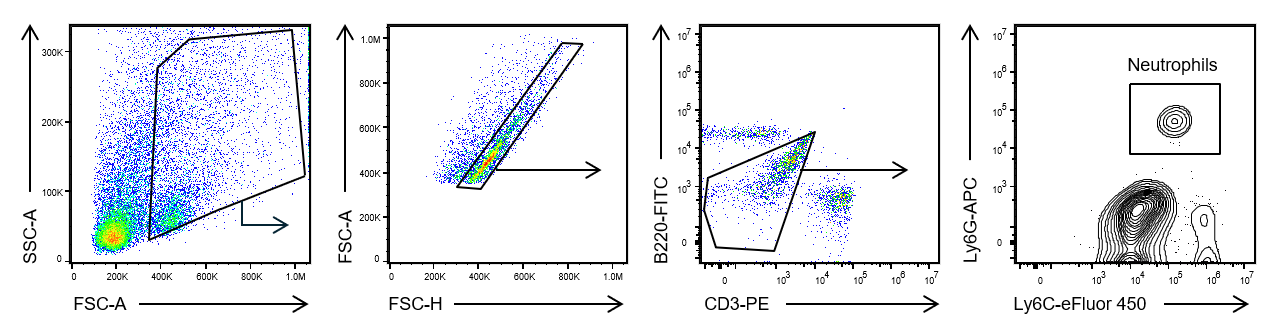

Supplement: S18 Fig — To identify neutrophil population, leucocytes were gated by forward and side scatter. Doublet signals are excluded by plotting forward scatter area versus forward scatter height. Then, B cells and T cells were excluded based on B220 and CD3 expression, respectively. Ly6C and Ly6G double-positive cells were identified as neutrophils. (TIF) [file ppat.1012776.s018.tif]
