# Supplementary material for: TNF-α exacerbates SARS-CoV-2 infection by stimulating CXCL1 production from macrophages
Source: PLoS Pathog. 2024 Dec 9;20(12):e1012776. doi: 10.1371/journal.ppat.1012776 (PMC11658697; doi:10.1371/journal.ppat.1012776)
Supplement: Attachment — Submitted filename: 241119 Response to Reviewers.pdf [file ppat.1012776.s022.pdf]

Part II – Major Issues: Key Experiments Required for Acceptance

Please use this section to detail the key new experiments or modifications of existing experiments that should be absolutely required to validate study conclusions.

Generally, there should be no more than 3 such required experiments or major modifications for a "Major Revision" recommendation. If more than 3 experiments are necessary to validate the study conclusions, then you are encouraged to recommend "Reject".

Reviewer #1: No.

Reviewer #2: The authors have provided explanation for the comments raised during previous iteration. However, the authors have failed to address majority of the concerns expressed by this reviewer.

Original Comment 1: Study implications- The authors were suggested to explain the implications of the current results to human WA and Delta SARS-CoV-2 infection. Although the authors mention that they included an explanation, those modifications do not discuss implications of the current results with the outcomes in humans.

This is a missed opportunity, since highlighting the differential role of IFN-I and Myd88 in Delta MA vs WA-MA virus infected mice and providing including implications to human infections would have informed clinicians and scientist alike about differential role of these signaling pathways in different SARS-CoV-2 variant infections. The authors could have also re-written the study to highlight that severe disease upon delta variant infection could be due to differential and pathogenic role of the above mentioned pathway.

We sincerely apologize for not fully understanding the original comment 1 from the reviewer last time. According to the reviewer's suggestion, we have now added three important studies to the Discussion section (lines 298-301 page 14), which report that some severe COVID-19 patients have deficiencies in TLR3, TLR7, or IFN signaling, or possess autoantibodies against type I IFNs (Refs #27-29). In addition, we included an explanation of how differences in the infectious doses of SARS-CoV-2 (a physiological low dose vs an experimental lethal dose), individuals with deficiencies in innate immune signals or those possessing autoantibodies against type I IFNs, and the differences between the ancestral

P80 and delta P80 viruses used in this study can result in host interferons having both protective roles and pathogenic aspects (lines 314-323 page 15). We hope these explanations clarify how this study contributes to the understanding of SARS-CoV-2 infections in humans

Original Comment 2: The authors were asked to provide rationale for developing a MA virus that causes severe disease in 6-week old mice as opposed to MA-virus by other labs that cause severe disease 12-20 week or older old mice. The justification of eliminating age as a factor and cellular senescence are not satisfactory, as 12-20 week mice are not old mice and they likely do not have senescent cells, unlike 20month old mice.
We appreciate the reviewer's insightful comment regarding the rationale for developing a mouse-adapted SARS-CoV-2 that causes severe disease in 6-week-old C57BL/6J mice. The primary reasons for this approach are: (1) 6-week-old mice are physiologically uniform, allowing for consistent experimental outcomes; (2) this model provides insights into the pathogenesis independent of age-related immune changes; (3) it serves as a robust platform for testing therapeutics and vaccines; (4) it complements existing models in older mice, addressing different scientific questions; and (5) it has potential clinical relevance by enabling studies on disease mechanisms and interventions applicable to younger populations. We hope this justification clarifies the value of this model. According to the reviewer’s suggestion, we have now added an explanation to clarify this point (lines 84-92 page 5).

Original Comment 3: No explanation provided to explain why IFN-I and Myd88 may cause severe disease upon delta MA infection compared to WA-MA infection.
We are grateful for your valuable feedback. We appreciate the insights provided by the reviewers, which have greatly enhanced the discussion. According to the reviewer’s suggestion, we have now added three possible explanations for how specific mutations observed in the Delta P80 virus contribute to MyD88/IFNAR1-mediated severe disease outcomes to the Discussion section. (lines 363-382 pages 17-18).

Original Comment 5: The authors were suggested to use TNF-/- mice or anti-TNF and anti-CXCL-1 mAb to show direct and endogenous role of these mediators in disease pathogenesis, as exogenous administration TNF, CXCL-1, and other

inflammatory mediators will likely have adverse outcomes. Therefore, it is critical to block/neutralize endogenous levels of these mediators to show their clinical relevance. However, the authors could not do these studies and cite expense associated with the neutralizing antibodies as key reason to not perform the studies. These antibodies (anti-TNA) are available through BioXcell and Leinco technologies at affordable rate.

First of all, we would like to sincerely thank the reviewer for sharing such valuable information. We were not aware that antibodies could be purchased at reasonable prices from these companies. According to the reviewer's suggestion, we have now tested the therapeutic effects of anti-TNF-α antibodies against lethal Delta P80 virus infection (please see Figure for reviewer below).

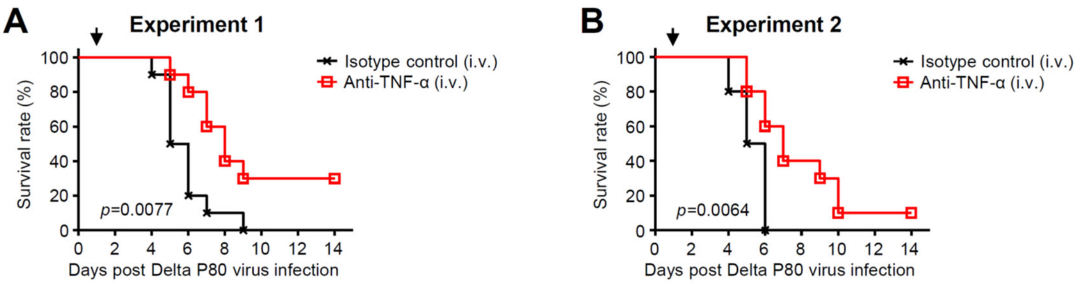

Figure for reviewer. Six-week-old C57BL/6 mice infected with 1 × 10^5 pfu of the Delta P80 virus were administered intravenously with isotype rat IgG (10 μg) or anti-TNF-α antibodies (10 μg) at 1-day p.i. (allow). Mortality was monitored for 14 days. These experiments were repeated twice (A and B).

We found that intranasal administration of anti-TNF-α antibodies has no effect on the survival rate of the Delta P80 virus-infected mice (please see new S15 Fig). In contrast, intravenous administration of anti-TNF-α antibodies to mice significantly improved survival after the Delta P80 virus infection compared to isotype control group. We have now added combined data in S16 Fig.

The authors instead use TAPI-2, a inhibitor of matrix matalloproteases (targets several MMPs) and TACE (targets TNF). The results obtained using TAPI-2 are not specific to TNF, and therefore the conclusions are not well justified. Moreover, TAPI-2 is given via IP route instead of IN route. Additionally, DNase could have several off target effects.

According to the reviewer's suggestion, we investigated whether intranasal administration of TAPI-2 improves the survival rate of the Delta P80 virus-infected mice. As in the experiments where anti-TNF-α antibodies were administered intranasally, intranasal administration of TAPI-2 has no effect on the survival rate

of the Delta P80 virus-infected mice. We have now added this data in S11 Fig.
