# Supplementary material for: TNF-α exacerbates SARS-CoV-2 infection by stimulating CXCL1 production from macrophages
Source: PLoS Pathog. 2024 Dec 9;20(12):e1012776. doi: 10.1371/journal.ppat.1012776 (PMC11658697; doi:10.1371/journal.ppat.1012776)
Supplement: Attachment — Submitted filename: 240925 Response to Reviewers.docx [file ppat.1012776.s021.docx]

**Part I - Summary**
Please use this section to discuss strengths/weaknesses of study, novelty/significance, general execution and scholarship.

Reviewer #1: Deguchi et al's manuscript "TNF-α exacerbates SARS-CoV-2-infection by stimulating CXCL-1 production from macrophages" describes 2 mouse adapted SARS-CoV-2 infection models and investigates the pathogenesis of SARS-CoV-2 infection based on these models. Their work provides potential tools for further identify the pathology of COVID-19. However, some substantial concerns need to be addressed.

Reviewer #2: This manuscript titled “TNF exacerbates SARS-CoV-2 infection by stimulating CXCL-1 production from macrophages” by Deguchi K et al. has two primary objectives: a) to develop a reliable mouse-adapted (MA)-virus that causes severe disease in commonly used young C57BL/6 mice, and b) to investigate the basis for SARS-CoV-2-induced severe disease using the novel MA virus. Here, the authors examine underlying basis for SARS-CoV-2-induced cytokine storm and severe disease using MA SARS-CoV-2 viruses derived from different human variants. The authors show that high-passaged (p80) MA SARS-CoV-2 derived from delta variant, but not the ancestral variant, caused lethal disease in young B6 mice, while P80 virus from both backgrounds caused severe disease in BALB/c and C3H mice. In young B6 mice, the P80 MA-delta virus replicated to high titers, caused lung pathology, and triggered a robust cytokine response. The authors also showed that Myd88 and IFN-I signaling were pathogenic, early IFN-β treatment protected, and the delayed IFN-β administration caused pathology in P80 MA-delta virus-infected B6 mice. Mechanistically, the IFN-I-induced TNF-mediated CXCL-1 response was associated with severe disease in P80 MA-delta virus-infected young B6 mice.

Strengths:
This is an interesting study wherein the authors have developed a novel MA virus on the Delta variant background for studying SARS-CoV-2 pathogenesis in B6 mice. The study also highlights the SARS-CoV-2 variant-specific role of IFN-I and Myd88 in cytokine storm and severe disease. Additionally, the work identifies the role of the TNF-CXCL-1 axis in causing severe lung inflammation and lethal pneumonia. However, there are several major concerns that diminish the enthusiasm for this work in its current form. Comments are listed below.

Weaknesses:
1. Study implications: It is now well-established that the lack of IFN-I and TLR3/7 signaling is associated with severe COVID19. As a result, significance and implications of the differential role of IFN-I and Myd88 in MA-ancestral and MA-delta virus-infected mice, and how these results correlate with COVID-19 outcomes in humans, are not clear, nor are well articulated. Specifically, it is unclear whether the loss of IFN and Myd88 signaling is associated with less severe disease in humans infected with the Delta variant.

We appreciate the reviewer’s comment. Despite our best efforts, we have been unable to find any information regarding the potential effects of IFN and MyD88 signaling deficiency on COVID-19 outcomes in humans infected with the Delta variant. According to the reviewer’s suggestion, we have now modified the discussion to clarify the significance of these findings using a mouse model of SARS-CoV-2 infection for human pathology (lines 286-298 page 14, lines 358-361 page 17).

2. MA viruses developed by different laboratories (including MA-10 by Baric lab and MA-30 by Perlman lab) are extensively used to study SARS-CoV-2 pathogenesis. Although these viruses cause mild disease in young 6-week B6 mice, these viruses (specifically MA30) cause lethal disease in adult (16-20 week or older) B6 mice. Therefore, the premise of having to develop a MA virus specifically to induce severe disease in 6-week mice is not justified. Additionally, 6-week mice, although extensively used, are too young for SARS-CoV-2 studies, a virus that causes mild disease in young individuals.

We thank the reviewer for pointing this out. According to the reviewer’s suggestion, we have now added an explanation to clarify this point (lines 84-88 page 5).

3. The differential disease outcomes following MA-ancestral and MA-delta virus infections are novel. However, it would be interesting to know if specific mutations observed in MA-delta contribute to IFN-I/Myd88 mediated severe disease outcomes.

We thank the reviewer for pointing this out. Despite our best efforts, we have been unable to find any information regarding the potential effects of the mutations observed in the Delta P80 virus on the interferon pathway. Although it is interesting to know if specific mutations observed in the Delta P80 virus contribute to interferon pathways and severe disease outcomes, we believe that demonstration of such mechanism is outside the scope of this current study and we wish to pursue this further in a separate future study.

4. The protective and detrimental roles of early and delayed IFN-β treatment, respectively, are well described for SARS-CoV, MERS-CoV, and SARS-CoV-2 infection by several investigators, making these observations less novel. However, the role of IFN-I mediated TNF signaling in CXCL1-induced lung pathology is novel and significant.

We are grateful for the reviewer's positive comments about our study. According to the reviewer’s suggestion, we have now modified the sentence to clarify the novel finding of this study (lines 41-42 page 2, lines 55-56 page 3).

5. Figure 7: CXCL-1 and TNF treatment enhanced disease severity in ancestral and delta P80 MA virus-infected mice. However, TAPI treatment marginally, albeit significantly, enhanced survival. These results show that perhaps using knockout mice or blocking TNF and CXCL-1 using specific monoclonal antibodies is a better approach compared to using an inhibitor.

We thank the reviewer for pointing this out. In this regard, we would like to respectfully bring to the reviewer's attention that the effect of TAPI-2 appears to be more pronounced in aged mice (see S11 Fig). The pronounced effect of TAPI-2 in aged mice may be explained by the fact that the aged mice exhibited a greater production of TNF than the younger mice (new S12 Fig). We have now included these data and explanation in the revised manuscript.

With respect to TNF knockout mice, we have been unable to set up experiments using deficient mice over the past two months. Recently, Bader and colleague have demonstrated that aged TNF KO mice exhibit resistance to lethal infection with SARS-CoV-2 N501Y P21 virus infection without affecting virus titers in the lung compared with aged WT mice (Bader et al. Proc Natl Acad Sci U S A. 2023) (see lines 316-318 page 15).

As for an experiment using TNF- or CXCL1-specific monoclonal antibodies, we conducted an experiment using DNase I as an alternative because it is expensive to purchase the amounts of antibodies needed for in vivo administration. Neutrophil extracellular traps digestion in vivo resulted in prolonged survival of mice after the Delta P80 virus infection (new Fig 7K). However, the effect of DNase I treatment is still marginal. We wish to resolve these issues in the future.

6. The authors postulate that TNF-mediated CXCL-1-induced neutrophils cause inflammation and pathology in delta-MA infected mice. However, no neutrophil data (FACS or histology) is available to support these conclusions.

We thank the reviewer for pointing this out. According to the reviewer’s suggestion, we have now tested CXCL1 production and neutrophil recruitment in the lung after intranasal administration of TNF-α. We showed that intranasal administration of TNF-α stimulated CXCL1 production and neutrophil recruitment into the lung tissue (see new Fig 7I and 7J). In addition, neutrophil extracellular traps digestion in vivo resulted in prolonged survival of mice after the Delta P80 virus infection (new Fig 7K). These data considerably strengthen our original claim that TNF-α exacerbates the Delta P80 infection by stimulating CXCL1 production from macrophages and neutrophil recruitment into the lung tissue. We have now included these data and explanation in the revised manuscript.

**Part II – Major Issues: Key Experiments Required for Acceptance**
Please use this section to detail the key new experiments or modifications of existing experiments that should be absolutely required to validate study conclusions.

Generally, there should be no more than 3 such required experiments or major modifications for a "Major Revision" recommendation. If more than 3 experiments are necessary to validate the study conclusions, then you are encouraged to recommend "Reject".

Reviewer #1: Major concerns

1 In this work, SARS-CoV-2 viruses were adapted in murine system by serial passages, which is expected to lead to new mutations as shown in Fig. 1 and 3. Authors need to provide further interpretations on these mutations, especially their clinical relevance. If these mutations are identified in SARS-CoV-2 variants circulating in human population as well, the value of these models will be significantly improved.

We thank the reviewer for pointing this out. According to the reviewer’s suggestion, we investigated whether these mutations were found in viruses circulating in humans and found two clinically relevant mutations. First, nsp9-T35I substitution (Orf1a T4175I), which was found in the Delta P80 virus, is a characteristic mutation of the currently circulating EG.5 and BA.2.86/JN.1 variants (Kaku Y et al. Lancet Infect Dis. 2024, Ip et al. EBioMedicine. 2024). Second, the E-T30I substitution, which was found in the ancestral P80, Delta P18, P29, P41, P61, and P80 viruses, was determined to be the second most frequent recurrently occurring mutation arising in persistent infections (Wilkinson et al. Virus Evol. 2022). Importantly, a single variant lineage, B.1.616 that is associated with high lethality (Fillâtre et al. Clin MIcrobiol Infect. 2022), does contain E-T30I as a lineage-defining mutation (Wilkinson et al. Virus Evol. 2022). We have now cited (references #21-24) and discussed these papers (lines 268-275 page 13)

2 In the pathogenesis investigation, ancestral P80 and delta P80 were used as moderate and lethal models, respectively, which need to be clarified to avoid confusions. Based on the severity caused by different viruses, the role of MyD88 and IFNAR1, as well as different treatment of type I IFN were used correspondingly. Again, these designs are understandable, but the generated data need to be interpreted separately because the pathology of COVID-19 caused by different variants, especially those carrying multiple mutations, could be distinct. In addition, the authors are encouraged to establish different severity model using the same virus by optimizing the infectious doses.

We thank the reviewer for pointing this out. According to the reviewer’s suggestion, we examined changes in body weight and survival in MyD88-deficient mice compared to wild-type mice. by reducing the challenge dose of Delta virus. However, our observations did not reveal any notable differences in body weight or survival rate between WT and MyD88 KO mice (see new S8 Fig). We have now replaced “SARS-CoV-2” with “the Delta P80 virus” to avoid this confusion (lines 224-228 page 11).

3 The author claim the critical role of CXCL-1 in TNF-induced inflammation but failed to provide direct evidence. In vivo TNF-dependent CXCL-1 expression by macrophage is warranted. Meanwhile, the underlying mechanisms contributing to CXCL-1-mediated inflammation (by recruiting neutrophil as shown in Fig. s4? If so, the neutrophil accumulation in affected lung need to be determined) need to be further identified.

We thank the reviewer for pointing this out. According to the reviewer’s suggestion, we have now tested CXCL1 production and neutrophil recruitment in the lung after intranasal administration of TNF-α. We showed that intranasal administration of TNF-α stimulated CXCL1 production and neutrophil recruitment into the lung tissue (see new Fig 7I and 7J). In addition, neutrophil extracellular traps digestion in vivo resulted in prolonged survival of mice after the Delta P80 virus infection (new Fig 7K). These data considerably strengthen our original claim that TNF-α exacerbates the Delta P80 infection by stimulating CXCL1 production from macrophages and neutrophil recruitment into the lung tissue.

Reviewer #2:

1. The differential disease outcomes following MA-ancestral and MA-delta virus infections are novel. However, it would be interesting to know if specific mutations observed in MA-delta contribute to IFN-I/Myd88 mediated severe disease outcomes.

We thank the reviewer for pointing this out. Despite our best efforts, we have been unable to find any information regarding the potential effects of the mutations observed in the Delta P80 virus on the interferon pathway. Although it is interesting to know if specific mutations observed in the Delta P80 virus contribute to interferon pathways and severe disease outcomes, we believe that

demonstration of such mechanism is outside the scope of this current study and we wish to pursue this further in a separate future study.

2. The protective and detrimental roles of early and delayed IFN-β treatment, respectively, are well described for SARS-CoV, MERS-CoV, and SARS-CoV-2 infection by several investigators, making these observations less novel. However, the role of IFN-I mediated TNF signaling in CXCL-1-induced lung pathology is novel and significant.

We are grateful for the reviewer's positive comments about our study. According to the reviewer’s suggestion, we have now modified the sentence to clarify the novel finding of this study (lines 41-42 page 2, lines 55-56 page 3).

3. Figure 7: CXCL-1 and TNF treatment enhanced disease severity in ancestral and delta P80 MA virus-infected mice. However, TAPI treatment marginally, albeit significantly, enhanced survival. These results show that perhaps using knockout mice or blocking TNF and CXCL-1 using specific monoclonal antibodies is a better approach compared to using an inhibitor.

We thank the reviewer for pointing this out. In this regard, we would like to respectfully bring to the reviewer's attention that the effect of TAPI-2 appears to be more pronounced in aged mice (see S11 Fig). The pronounced effect of TAPI-2 in aged mice may be explained by the fact that the aged mice exhibited a greater production of TNF than the younger mice (new S12 Fig). We have now included these data and explanation in the revised manuscript.

With respect to TNF knockout mice, we have been unable to set up experiments using deficient mice over the past two months. Recently, Bader and colleague have demonstrated that aged TNF KO mice exhibit resistance to lethal infection with SARS-CoV-2 N501Y P21 virus infection without affecting virus titers in the lung compared with aged WT mice (Bader et al. Proc Natl Acad Sci U S A. 2023) (see lines 316-318 page 15).

As for an experiment using TNF- or CXCL1-specific monoclonal antibodies, we conducted an experiment using DNase I as an alternative because it is expensive to purchase the amounts of antibodies needed for in vivo administration. Neutrophil extracellular traps digestion in vivo resulted in prolonged survival of mice after the Delta P80 virus infection (new Fig 7K). However, the effect of DNase I treatment is still marginal. We wish to resolve these issues in the future.

4. The authors postulate that TNF-mediated CXCL-1-induced neutrophils cause inflammation and pathology in delta-MA infected mice. However, no neutrophil data (FACS or histology) is available to support these conclusions.

We thank the reviewer for pointing this out. According to the reviewer’s suggestion, we have now tested CXCL1 production and neutrophil recruitment in the lung after intranasal administration of TNF-α. We showed that intranasal administration of TNF-α stimulated CXCL1 production and neutrophil recruitment into the lung tissue (see new Fig 7I and 7J). In addition, neutrophil extracellular traps digestion in vivo resulted in prolonged survival of mice after the Delta P80 virus infection (new Fig 7K). These data considerably strengthen our original claim that TNF-α exacerbates the Delta P80 infection by stimulating CXCL1 production from macrophages and neutrophil recruitment into the lung tissue. We have now included these data and explanation in the revised manuscript.

5. The authors show increased virus titers in MA-delta (p80) infected mice compared to MA-ancestral delta (p80). As shown, it is not obvious whether severe disease upon P80-MA-delta virus-infected mice is due to high virus titers or virus-induced inflammation or both. A side-by-side comparison of lung inflammation in MA-delta and ancestral-MA virus-infected mice/lungs is required to support these conclusions.

We thank the reviewer for pointing this out. In this regard, we would like to respectfully bring to the reviewer's attention that the MyD88 or IFNAR1 KO mice had improved survival relative to WT mice following the Delta P80 virus infection (Fig 5C-5F) without affecting the virus titer at 3 days p.i. (Fig 5G and 5H). To address the reviewer's question regarding the inflammatory responses, we measured cytokines production in lung washes after the ancestral or Delta P80 virus infection. We found that the virus-induced proinflammatory cytokines (IFN-α, IFN-β, IL-6, and TNF-α) or chemokine (CXCL1) were significantly elevated in lung of the Delta P80 virus-infected mice compared with those of ancestral P80 virus infected mice (new Fig 4F-4J). These data and cell tropism analysis (see below) suggest that enhanced viral proliferation in the lung and increased infection of alveolar macrophages and dendritic cells with the Delta P80 virus can cause severe inflammation, which may increase disease severity.

6. It would be interesting to know whether MA-delta and ancestral-MA viruses have differential cell tropism within the lungs and in extrapulmonary tissues. Perhaps this would also explain the basis for differential outcomes.

We appreciate the reviewer for this insightful suggestion. According to the reviewer’s suggestion, we have now carried out two separate sets of experiments to clarify this point. First, we examined the possibility that the Delta P80 virus infects tissues other than the lung in mice. However, we were unable to detect viral RNA in the brain, heart, liver or kidney of mice infected with the ancestral or Delta P80 viruses (see new Fig 4L and 4M). Second, we examined whether the ancestral and Delta P80 viruses have differential cell tropism within the lungs. While the primary target of these two variants was the CD45.2 negative epithelial cells in the lung (new Fig 4K and S2 Fig), the Delta P80 virus also infected a large number of alveolar macrophages and dendritic cells in the lung tissue (new S3-S7 Fig). These results may explain why the Delta variant causes severe inflammation in vivo. We have now included these data and added explanation in the revised manuscript (lines 153-163 page 8).

**Part III – Minor Issues: Editorial and Data Presentation Modifications**
Please use this section for editorial suggestions as well as relatively minor modifications of existing data that would enhance clarity.

Reviewer #1: Minor concerns
The introduction need to be polished. Some information, such as "experimental techniques for tracheal surgery are required for intratracheal inoculation of the AAV vector" is not accurate because AAV transfection can be accomplished well by intranasal inoculation.

We thank the reviewer for pointing this out. According to the reviewer’s suggestion, we decided to delete the sentence "but experimental techniques for tracheal surgery are required for intratracheal inoculation of the AAV vector " (line 66 page 4).

Reviewer #2:

1. Introduction lacks a clear rationale. It appears that the goal of the study is to develop an MA virus that causes severe disease in 6-week-old B6 mice.

We thank the reviewer for pointing this out. According to the reviewer’s suggestion, we have now added an explanation to clarify this point (lines 84-88 page 5).

2. The rationale for using aged mice to initially adapt the viruses is not clear. While most of the advanced variants do bind to murine ACE2, the ACE2 binding ability of the ancestral variant (spike) used in this study is not provided.

We thank the reviewer for pointing this out. According to the reviewer’s suggestion, we have now added an explanation to clarify this point (lines 101-102 page 6).

3. Figure 2 and elsewhere: the authors estimate titers in BALF. Why not estimate titers in lungs?

I have been measuring virus titers in BALF for over 20 years, since I started studying influenza virus research as a graduate student (Ichinohe et al. J Virol. 2005). Since viral infection changes the weight of the lungs depending on the degree of inflammation (see Fig 4D), I wonder if the virus titers (i.e. pfu/g) might be affected by the lung weight.

4. Figure 4: The authors rely on lung edema and lung weight for inflammation studies. A thorough histopathological and flow cytometry evaluation of lungs/lung cells from MA-delta and ancestral-MA virus-infected mice is critical to establish lung inflammation.

We thank the reviewer for pointing this out. According to the reviewer’s suggestion, we have now examined the neutrophil recruitment into the lung tissue after the ancestral or Delta P80 virus infection (see new S1 Fig).

5. Lines 304-317: The discussion is not overtly relevant and needs to be more aligned with the study objectives to support the results.

We thank the reviewer for pointing this out. According to the reviewer’s suggestion, we decided to exclude the paragraph and rewrote the discussion (lines 334-356 pages 16-17).

6. Discuss the relevance of these findings with human COVID-19 following ancestral and delta variant infections.

We thank the reviewer for pointing this out. According to the reviewer’s suggestion, we added a discussion to clarify the relevance of current study with human COVID-19. First, we added to the discussion that two mutations observed in the ancestral and delta P80 viruses are characteristic mutations of the currently circulating EG.5 and BA.2.86/JN.1 variants (lines 268-275 page 13). Second, we added a discussion that while interferon signaling contributes to hots defense to SARS-CoV-2 infection, it is also related to the severity of the virus infection, depending on the timing of interferon responses and challenge doses across the experimental condition (lines 293-300 pages 14-15). Because decreased IFN and elevated proinflammatory cytokines are a common characteristic of the innate immune system in older humans, we added to the discussion that our results imply a possible effect of SARS-CoV-2-induced proinflammatory cytokines in severity of the diseases in elderly people (lines 302-303 page 15). Finally, we discussed the relevance of these findings with human COVID-19 following ancestral and delta variant infections (lines 334-356 pages 16-17).
